# Prenatal Detection of Trisomy 2: Considerations for Genetic Counseling and Testing

**DOI:** 10.3390/genes14040913

**Published:** 2023-04-14

**Authors:** Olga E. Talantova, Alla S. Koltsova, Andrei V. Tikhonov, Anna A. Pendina, Olga V. Malysheva, Olga A. Tarasenko, Elena S. Vashukova, Elena S. Shabanova, Arina V. Golubeva, Olga G. Chiryaeva, Andrey S. Glotov, Olesya N. Bespalova, Olga A. Efimova

**Affiliations:** 1 D.O. Ott Research Institute of Obstetrics, Gynecology and Reproductology, Mendeleevskaya Line, 3, St. Petersburg 199034, Russia; 2 Faculty of Biology, Department of Genetics and Biotechnology, St. Petersburg State University, Universitetskaya emb., 7/9, St. Petersburg 199034, Russia

**Keywords:** trisomy 2, mosaicism, invasive prenatal diagnosis, non-invasive prenatal diagnosis (NIPT), ultrasound markers of chromosomal abnormalities, low-level mosaicism

## Abstract

We report on the case of prenatal detection of trisomy 2 in placental biopsy and further algorithm of genetic counseling and testing. A 29-year-old woman with first-trimester biochemical markers refused chorionic villus sampling and preferred targeted non-invasive prenatal testing (NIPT), which showed low risk for aneuploidies 13, 18, 21, and X. A series of ultrasound examinations revealed increased chorion thickness at 13/14 weeks of gestation and fetal growth retardation, a hyperechoic bowel, challenging visualization of the kidneys, dolichocephaly, ventriculomegaly, increase in placental thickness, and pronounced oligohydramnios at 16/17 weeks of gestation. The patient was referred to our center for an invasive prenatal diagnosis. The patient’s blood and placenta were sampled for whole-genome sequencing-based NIPT and array comparative genomic hybridization (aCGH), respectively. Both investigations revealed trisomy 2. Further prenatal genetic testing in order to confirm trisomy 2 in amniocytes and/or fetal blood was highly questionable because oligohydramnios and fetal growth retardation made amniocentesis and cordocentesis technically unfeasible. The patient opted to terminate the pregnancy. Pathological examination of the fetus revealed internal hydrocephalus, atrophy of brain structure, and craniofacial dysmorphism. Conventional cytogenetic analysis and fluorescence in situ hybridization revealed chromosome 2 mosaicism with a prevalence of trisomic clone in the placenta (83.2% vs. 16.8%) and a low frequency of trisomy 2, which did not exceed 0.6% in fetal tissues, advocating for low-level true fetal mosaicism. To conclude, in pregnancies at risk of fetal chromosomal abnormalities that refuse invasive prenatal diagnosis, whole-genome sequencing-based NIPT, but not targeted NIPT, should be considered. In prenatal cases of trisomy 2, true mosaicism should be distinguished from placental-confined mosaicism using cytogenetic analysis of amniotic fluid cells or fetal blood cells. However, if material sampling is impossible due to oligohydramnios and/or fetal growth retardation, further decisions should be based on a series of high-resolution fetal ultrasound examinations. Genetic counseling for the risk of uniparental disomy in a fetus is also required.

## 1. Introduction

Prenatal detection of rare chromosomal abnormalities may present distinct challenges for interpreting test results and adequate genetic counseling. In first-trimester pregnancies, fetal chromosomal abnormalities can be detected by non-invasive prenatal testing (NIPT) or invasive prenatal diagnosis—methods based on the analysis of the chorion. In most cases, typically lethal trisomies found in chorionic villi need further testing of the fetus in order to differentiate confined placental mosaicism from true fetal mosaicism.

Complete trisomy 2 is a lethal chromosomal abnormality. It is registered in ~1–2% of first-trimester miscarriages [1,2,3]. Prenatal trisomy 2 detection is not frequent in prenatal diagnosis and typically represents cases of mosaicism confined to the placenta. True fetal trisomy 2 mosaicism is associated with multiple system abnormalities in fetuses/newborns, resulting in an unfavorable prognosis [4,5,6,7,8]. Due to low levels of mosaicism, this condition may present a challenge for prenatal genetic diagnosis.

This paper reports a case of prenatally detected trisomy 2 and presents our conclusions regarding the most optimal genetic counseling and testing algorithm in such cases.

## 2. Detailed Case Description

A 29-year-old G2P1 woman with a desired pregnancy was followed up by a local women’s health clinic. Her first desired pregnancy resulted in the birth at term of a healthy and normal-weight boy. The patient reported no chronic conditions or exposure to occupational hazards. She was married to a non-consanguineous partner. Both spouses reported unremarkable family histories.

At 11 weeks and 5 days of gestation, the patient had her first-trimester ultrasound screening. The ultrasound examination revealed fetal crown-rump length measuring 54.0 mm, nuchal translucency of 1.90 mm, and the presence of fetal nasal bone. Neither congenital malformations nor first-trimester ultrasound markers of chromosomal pathology were detected. Biochemical screening for chromosomal abnormalities reported a free β-hCG level of 3.490 MoM and a PAPP-A level of 0.366 MoM. The individual trisomy 21 risk produced a high estimate of 1:24.

The patient refused invasive prenatal diagnosis by chorionic villus sampling (CVS). Instead, she preferred targeted NIPT for trisomies 13, 18, 21, and monosomy X. The results for all the tested aneuploidies were reported as low probability.

A follow-up ultrasound examination at 13 weeks and 6 days of gestation revealed increased chorion thickness. The rest of the fetal morphology was normal. An additional elective ultrasound at 16 weeks and 6 days of gestation exhibited stage 1 symmetric fetal growth retardation, a hyperechoic fetal bowel, challenging visualization of the kidneys, a manifest increase in placental thickness, and pronounced oligohydramnios.

**Figure 1:**
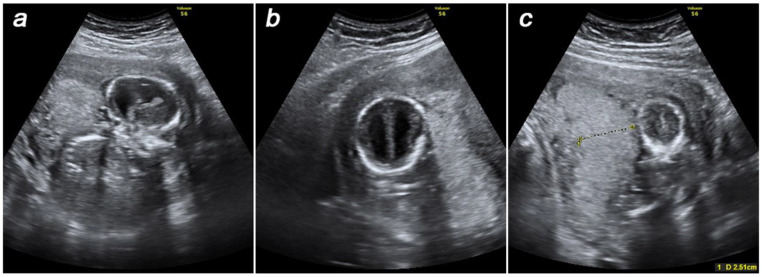
Prenatal ultrasound findings at 16–17 weeks of gestation: dolichocephaly (**a**), ventriculomegaly (**b**), thick placenta (**c**).

At 16–17 weeks of gestation, the patient presented these findings to the genetic counselors of our center at the D.O. Ott Research Institute of Obstetrics, Gynecology, and Reproductology where she was referred for prenatal karyotyping. Prior to the invasive prenatal diagnosis procedure, an ultrasound examination was performed to produce new findings of fetal dolichocephaly and ventriculomegaly (Figure 1a,b). Considering pronounced oligohydramnios and specific placental anatomy (Figure 1c), as well as fetal growth retardation, neither amniocentesis nor cordocentesis was technically feasible, making placental biopsy the technique of choice.

The obtained amount of placental villi was not enough for conventional karyotyping. The biopsy sample was therefore analyzed by array comparative genomic hybridization (aCGH), which allowed for the detection of trisomy of chromosome 2.

Prior to the invasive diagnostic procedure, maternal peripheral blood was sampled for whole-genome sequencing-based NIPT. The NIPT result showed a high risk for trisomy 2.

Due to multiple fetal anomalies and a confirmed chromosomal abnormality of the placenta, the patient and her spouse opted to terminate the pregnancy. Medical termination of the pregnancy was performed at the gestational age of 20 weeks. Fetal tissues and placenta were sampled for genetic diagnosis verification.

## 3. Laboratory Investigations and Diagnostic Tests 

### 3.1. Invasive and Non-Invasive Prenatal Genetic Investigations

**Figure 2:**
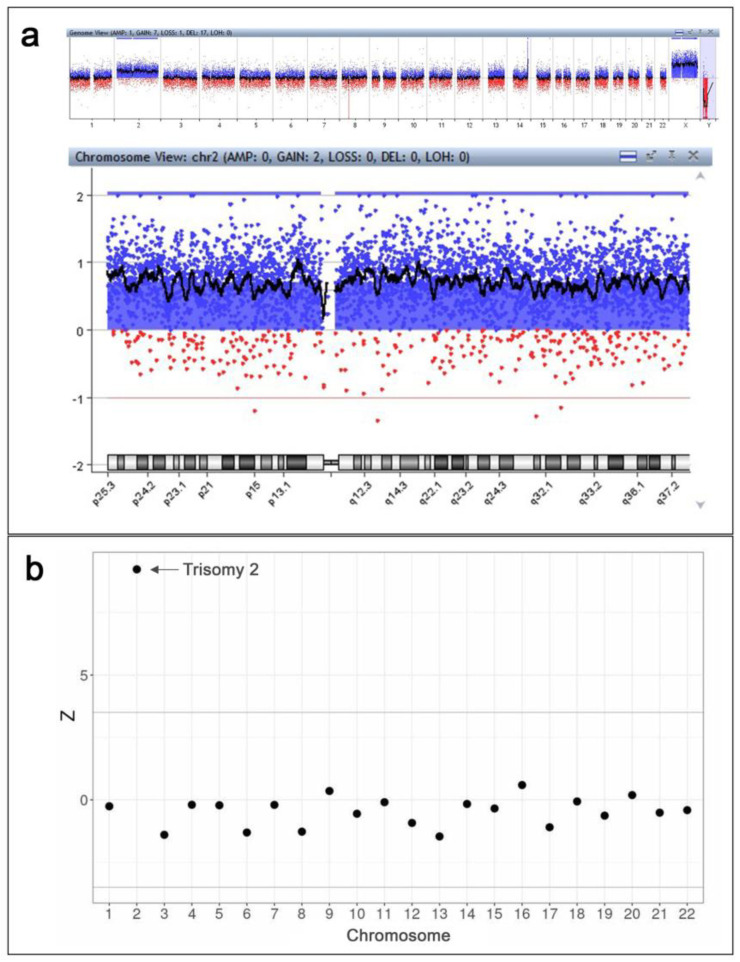
The results of invasive and non-invasive prenatal genetic investigations. (**a**) The results of CVS analysis by aCGH (Agilent CytoGenomics v.5.0.2.5): a complete trisomy 2 and a small deletion in 8p classified as a benign copy number variation (bCNV) are detected. The observed picture in 14q region is not a duplication, but a result of non-pathogenic deletion in a reference genome provided by the manufacturer (Agilent). (**b**) The results of whole-genome sequencing-based NIPT: a high risk of trisomy 2 is detected.

Invasive prenatal diagnosis was performed by placental biopsy and subsequent aCGH (G5963A 8 × 60, Agilent) according to the manufacturer’s protocol. The aCGH revealed complete trisomy 2 and a small deletion in 8p classified as a benign copy number variation (bCNV) (Figure 2a). Thus, the aCGH results were as follows: arr[GRCh37] (2)x3,8p11.22(39234992_39386158)x1.

Immediately prior to placental biopsy, the patient’s blood sample was collected into an EDTA-K2 tube for whole-genome sequencing-based NIPT according to the previously used protocol with minor modifications [9,10]. No later than 4 h after the blood draw, the blood sample underwent centrifugation 2000× *g* at +4 °C for 10 min. Blood plasma was then collected and centrifuged at 16,000× *g* for another 10 min at +4 °C. MagMAX Cell-Free DNA Isolation Kit (Thermo Fisher Scientific Inc., Waltham, MA, USA) was used to isolate plasma DNA. A DNA library was prepared using the Ion Plus Fragment Library Kit (Thermo Fisher Scientific Inc.). The Ion 540 Kit-Chef and Ion 540 Chips (Thermo Fisher Scientific Inc.) were used for automated template preparation and library construction. The Ion Torrent S5 system (Thermo Fisher Scientific Inc.) enabled sequencing workflows. The sequencing reads were filtered by mapping quality (>10) and read length (70–130 bp) using SAMtools [11]. The GRCh37 human genome assembly was used as a reference. The R SeqFF software was applied to identify fetal sex and estimate the fetal DNA fraction [12]. The regression-based Z-score and GC-content normalization algorithms were applied using NIPTeR, an open-source R package, to judge the risk threshold for fetal aneuploidy [13]. The analysis of fetal circulating DNA in the patient’s blood plasma exhibited a high risk of trisomy 2 (Figure 2b). 

### 3.2. Pathological and Histological Examination of the Fetus and Placenta after Pregnancy Termination

Pathological examination of the fetus revealed congenital malformation, including internal hydrocephalus with accompanying atrophy of brain structure and craniofacial dysmorphism (including a high, prominent forehead, a wide nasal arch, an upturned nose, and hypertelorism). 

Histopathological assessment of the placenta revealed focal purulent chorio-deciduitis and chronic placental insufficiency with evidence of dissociated villous maturation disorder and disrupted chorionic branching.

### 3.3. Genetic Characterization of the Fetus and Placenta after Pregnancy Termination

Fetal tissues were sampled to verify the prenatal genetic diagnosis. Tissues of the placenta, embryonic liver, lung, brain, heart, uterus, and femoral muscle were collected for cytogenetic and molecular cytogenetic characterization. Each specimen was split into two samples: one uncultured sample was used for “direct” (without culturing) cytogenetic preparations of interphase nuclei (for protocol, see [14]); the other was maintained in long-term cultures for further conventional karyotyping of QFH/AcD-banded chromosomes using previously described protocols [15,16,17].

The preparations of interphase nuclei from uncultured specimens of the placenta, fetal liver, lung, brain, heart, uterus, and femoral muscle were studied using fluorescence in situ hybridization (FISH) with DNA probes specific to the centromeric region of chromosome 2 (Vysis CEP 2, Abbott Molecular) and to the 2p24 region (Vysis N-MYC, Abbott Molecular). The chromosome 2 copy number in every interphase nucleus was estimated by calculating CEP 2 and N-MYC FISH signals. No fewer than 1000 interphase nuclei were studied in every specimen. Placental tissue showed chromosome 2 mosaicism with a prevalence of trisomic clone: trisomy 2 was detected in 83.2% of interphase nuclei vs. 16.8% of interphase nuclei showing disomy 2. In contrast, all the specimens of fetal tissue and organs showed a major proportion of interphase nuclei with two chromosome 2 copies; interphase nuclei with trisomy 2 did not exceed 0.6% in liver, lung, and heart tissue and were absent in the brain, uterus, and femoral muscle.

**Figure 3:**
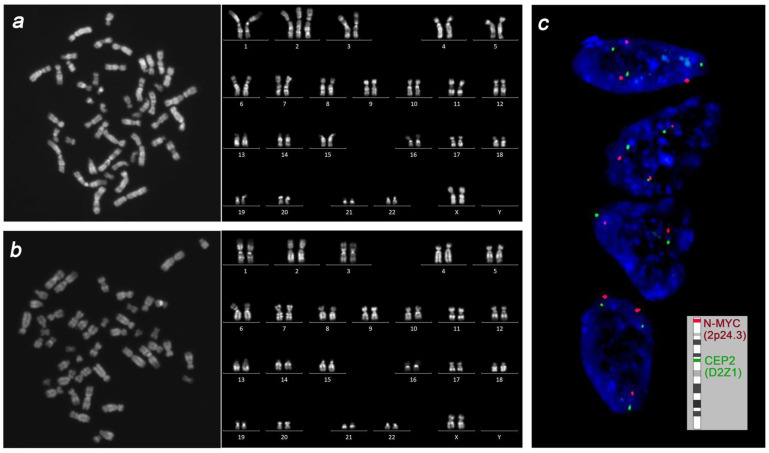
The cytogenetic picture in placenta and fetus. Conventional karyotyping revealed prevalence of cells with trisomy 2 in placenta (**a**) and normal karyotype in femoral muscle cells (**b**). Interphase FISH for chromosome 2 in uncultured specimens of the placenta and fetal tissues revealed prevalence of trisomic over disomic nuclei in placenta (**c**) and a low-level mosaicism for trisomy 2 in fetal liver, lung, and heart tissue.

In long-term cultures, cell growth was observed only in the placenta and fetal femoral muscle. Karyotyping yielded a mosaic placental karyotype, with the majority of cells displaying chromosome 2 trisomy: 47,XX,+2[24]/46,XX[9] (Figure 3). Fetal cells of the femoral muscle showed a normal karyotype: 46,XX[15]. Thus, cytogenetic and molecular cytogenetic examination revealed discordance in the proportion of disomic and trisomic for chromosome 2 cells in the placenta and fetal tissues.

Considering that observed mosaicism could result from trisomy rescue, testing for uniparental disomy (UPD) was performed. A quantitative fluorescent polymerase chain reaction (QF-PCR) assay was used to analyze the DNA from maternal peripheral blood and DNA from fetal tissues. Paternal DNA was not available for the analysis, which posed limitations for the interpretation of the results. Shortly, DNA was extracted by phenol–chloroform, and multiplex PCR with primers (Table 1) specific to STR-markers located in different loci of chromosome 2 (D2S272, D2S273, D2S278, and D2S1248) was performed. The analysis of the fluorescent PCR products was conducted by capillary electrophoresis using the ABI 3100-Avant Genetic Analyzer (Applied Biosystems, Waltham, MA, USA).

The amplified maternal DNA fragments of the D2S272 STR marker were 189 bp and 223 bp in length, while fetal DNA fragments had the size of 189 bp and 213 bp. The amplification of the D2S273 STR marker resulted in maternal amplified fragments being 238 bp and 250 bp in length, while those of fetal DNA were 238 bp in length. Maternal and fetal DNA fragments in the D2S278 STR marker were 286/300 bp and 286/297 bp, respectively. The size of maternal fragments in the D2S1248 STR marker was 343 bp and 360 bp, and that of fetal fragments was 343 bp and 382 bp in length (Table 2). Overall, the pattern of STR-marker inheritance advocates for the biparental inheritance of chromosome 2 in fetal tissues.

## 4. Discussion

An extra copy of chromosome 2 in the fetal karyotype is incompatible with full-term development and birth. In the case of mosaicism, however, such karyotypically abnormal fetuses have a more favorable predicted vitality. A few cases describing babies born with mosaic trisomy 2 have been reported so far [4,5,6,7,8]. All of the newborns, including those with low-level mosaicism, exhibited severe multiple defects of different systems—an issue that should be considered in cases of trisomy 2 prenatal detection and further genetic counseling.

Mosaic trisomy 2 is detected in 0.5–1.2/1000 prenatal tests using cytogenetic analysis of chorionic villi [4,18,19]. The indications for CVS often include changes in maternal plasma proteins at the first trimester combined screening test [20,21,22], which allows for early trisomy 2 detection either in the first or early second trimester. The presented clinical case exhibited changes in the first-trimester biochemical screening parameters (PAPP-A = 0.366 MoM and β-CGH = 3.49 MoM) and a high trisomy 21 risk estimate (1:24) that required the patient to refer for genetic counseling. However, considering the patient’s rejection of CVS and the unremarkable results of targeted NIPT, the patient was repeatedly recommended to undergo invasive prenatal diagnostic testing only at 16/17 weeks, once ultrasound markers of fetal chromosomal disease had been detected.

Specific chromosomal abnormalities, including trisomy 2, may arise spontaneously in chorionic and amniotic cell cultures. However, this scenario was excluded in our patient considering that the patient had undergone aCGH for prenatal diagnosis—the method that does not require placental villous tissue culture. Evidence suggests that a significant number of chorionic trisomy 2 cases represent confined placental mosaicism [19,23]. The latter cases can be associated with favorable pregnancy outcomes [23,24], except for trisomy 2, which causes disorders in placental function and significant intrauterine growth restriction (IUGR) [25,26,27,28]. Even in such a clinical setting, however, prenatal ultrasound would not recognize pronounced anomalies in any of the fetal systems. In contrast, most cases of true trisomy 2 mosaicism report evidence of specific fetal anomalies, including ventriculomegaly, congenital heart defect, and facial dysmorphism [29,30,31,32]. In our patient, the number of fetal abnormalities diagnosed by ultrasound—IUGR, ventriculomegaly, oligohydramnios—increased with gestation age. Clinical manifestations also included a markedly thick chorion and, then, thick placenta.

In the reported case, fetal ultrasound markers allowed for the suspicion of true trisomy 2 mosaicism. Prenatal cytogenetic confirmation was challenged by oligohydramnios and the impossibility of amniocentesis. IUGR made cordocentesis highly questionable as well. Considering the risk of a karyotypically abnormal fetus estimated by ultrasound examination and trisomy 2 evidence in placental cells (aCGH and whole-genome sequencing-based NIPT), the patient decided to terminate pregnancy with a follow-up genetic test of fetal tissues.

Pathological examination of the fetus confirmed severe developmental abnormalities. The cytogenetic investigation showed evidence of trisomy 2 mosaicism in the placenta and low-level trisomy 2 mosaicism in the fetal liver, lung, and heart. These findings are generally consistent with the data published by other authors, who also reported severe physical abnormalities despite small numbers of trisomy 2 cells. Robinson et al. reported 0–7% of cells trisomic for chromosome 2 across different tissues of the 23-week-old fetus with dysmorphic features, absence of gall bladder, cystic left kidney, a 13th left rib, and unilateral talipes [29]. A 2.5-year-old girl with a history of multiple congenital anomalies, including diaphragmatic hernia, duodenal atresia, intestinal malrotation, microcephaly, atrial and ventricular septal defect, developmental delay, hypotonia, and seizures, presented 3% mosaicism for trisomy 2 in fibroblasts [6]. In the study by Sago et al., 4% of cells trisomic for chromosome 2 were found in hepatic biopsy cultured fibroblasts, while blood, skin biopsy, and intestinal epithelium cells showed a normal karyotype in a male newborn with severe growth retardation, hydronephrosis, ureteral reflux, abnormal gastrointestinal motility, necrotizing enterocolitis, and ascites [4].

True trisomy 2 mosaicism might be the result of trisomy rescue in a trisomic conceptus, which implies the risk of maternal or paternal UPD in disomic cells. A few publications report evidence of chromosome 2 UPD diagnosed in fetuses with mosaic trisomy 2 in chorionic/placental cells or amniocytes [18,33,34,35]. In our case, UPD was excluded, suggesting that the observed clinical manifestations were caused by an extra copy of chromosome 2 in some placental and fetal cells.

A special matter for discussion is the role of NIPT—a method increasingly adopted as a screening tool in recent years—in the prenatal detection of trisomy 2 and other rare aneuploidies. Current studies suggest that different kinds of NIPT can detect rare aneuploidies, monogenic diseases, submicroscopic deletions or duplications, as well as the most common trisomies [36,37,38,39,40]. These studies, along with our own experience presented in this case report, strongly suggest that NIPT is able to effectively detect a high risk of trisomy 2 in chorionic/placental cells. Given that the procedure implies screening rather than diagnostic evaluation, NIPT should not be considered in cases where invasive prenatal testing is indicated. However, if a patient with no contraindications to invasion (risk of pregnancy termination or acute inflammatory reactions) refuses invasive diagnosis in favor of NIPT, the ultimate objective for genetic counselors is to properly inform the patient regarding individual risks and limitations associated with various kinds of NIPT. In our opinion, in the case of the revealed biochemical risk with no specific ultrasound markers of fetal chromosomal abnormalities and the patient’s strict refusal to undergo invasive prenatal diagnosis, targeted NIPT should never be considered, suggesting whole-genome sequencing-based NIPT as the only option. Otherwise, a potentially negative targeted NIPT result would have eventually exacerbated the delay of further diagnostic procedures, as it happened in the case of our patient.

Overall, the described clinical case as well as the data published by other authors allow one to conclude that whole-genome sequencing-based NIPT is a reliable tool to assess the risk of trisomy 2 in chorionic/placental cells. Once trisomy 2 in chorionic/placental tissue is confirmed, true trisomy 2 mosaicism shall be distinguished from placental confined trisomy 2 mosaicism using cytogenetic analysis of amniotic fluid cells as the most optimal technique. However, oligohydramnios—a common clinical manifestation of trisomy 2—may render amniocentesis unfeasible. The technical feasibility of cordocentesis may also be questionable due to fetal growth retardation. Therefore, further decisions should be based on the evidence of a high-resolution fetal ultrasound examination. In cases of pregnancy prolongation, genetic counseling for the risk of UPD in a fetus and associated diagnostic procedures are required. We believe that the reported data provide valuable knowledge that may be useful both for making decisions regarding NIPT and for genetic counseling in cases of trisomy 2 prenatal detection.

## Figures and Tables

**Table 1:** Primers used for multiplex QF-PCR testing of STR markers located in chromosome 2.

STR Marker	Primer
D2S272	F‒ 6-FAM- GGC CAG GTT GAA GAA CAG G R‒ TGG CTT GGG GAT TAT TGT CT
D2S273	F‒ ROX- AAA ATT GTG GTG GTG AAT GCT R‒ CAT CGT TTT CAG TTT GAG AGA GA
D2S278	F‒ 6-FAM- GAG CCC AGG GTG AAA GAA G R‒ AAT CAC ATG CGT CAA CTC CT
D2S1248	F‒ ROX- CCA TCC TCA GTA ACC CCT AC R‒ GCT CCC ATA CTC TCA CTT GC

**Table 2:** Maternal and fetal STR markers selected for uniparental disomy (UPD) testing.

	D2S272	D2S273	D2S278	D2S1248
Mother	**189–**223	**238**	**286–**300	**343–**360
Fetus	**189–**213	**238–**250	**286–**297	**343–**382

## Data Availability

The data will be available by contacting the corresponding author.

## References

[1] Pendina A.A., Efimova O.A., Chiryaeva O.G., Tikhonov A.V., Petrova L.I., Dudkina V.S., Sadik N.A., Fedorova I.D., Galembo I.A., Kuznetzova T.V. (2014). A Comparative Cytogenetic Study of Miscarriages after IVF and Natural Conception in Women Aged under and over 35 Years. J. Assist. Reprod. Genet..

[2] Soler A., Morales C., Mademont-Soler I., Margarit E., Borrell A., Borobio V., Muñoz M., Sánchez A. (2017). Overview of Chromosome Abnormalities in First Trimester Miscarriages: A Series of 1,011 Consecutive Chorionic Villi Sample Karyotypes. Cytogenet. Genome Res..

[3] Pylyp L.Y., Spynenko L.O., Verhoglyad N.V., Mishenko A.O., Mykytenko D.O., Zukin V.D. (2018). Chromosomal Abnormalities in Products of Conception of First-Trimester Miscarriages Detected by Conventional Cytogenetic Analysis: A Review of 1000 Cases. J. Assist. Reprod. Genet..

[4] Sago H., Chen E., Conte W.J., Cox V.A., Goldberg J.D., Lebo R.V., Golabi M. (1997). True Trisomy 2 Mosaicism in Amniocytes and Newborn Liver Associated with Multiple System Abnormalities. Am. J. Med. Genet..

[5] Gupta S., Shah S., Mcgaw A., Mercado T., Zaslav A.-L., Tegay D. (2007). Trisomy 2 Mosaicism in Hypomelanosis of Ito. Am. J. Med. Genet. A.

[6] Mihci E., Velagaleti G.V.N., Ensenauer R., Babovic-Vuksanovic D. (2009). The Phenotypic Spectrum of Trisomy 2: Report of Two New Cases. Clin. Dysmorphol..

[7] Hsu L.Y., Yu M.T., Neu R.L., Van Dyke D.L., Benn P.A., Bradshaw C.L., Shaffer L.G., Higgins R.R., Khodr G.S., Morton C.C. (1997). Rare Trisomy Mosaicism Diagnosed in Amniocytes, Involving an Autosome Other than Chromosomes 13, 18, 20, and 21: Karyotype/Phenotype Correlations. Prenat. Diagn..

[8] Prontera P., Stangoni G., Ardisia C., Rogaia D., Mencarelli A., Donti E. (2011). Trisomy 2 Mosaicism with Caudal Dysgenesis, Hirschsprung Disease, and Micro-Anophthalmia. Am. J. Med. Genet. A.

[9] Ivashchenko T.E., Vashukova E.S., Kozyulina P.Y., Dvoynova N.M., Talantova O.E., Koroteev A.L., Pendina A.A., Tikhonov A.V., Chiryaeva O.G., Petrova L.I. (2019). Noninvasive Prenatal Testing Using Next Generation Sequencing: Pilot Experience of the D.O. Ott Research Institute of Obstetrics, Gynecology and Reproductology. Russ. J. Genet..

[10] Pendina A.A., Shilenkova Y.V., Talantova O.E., Efimova O.A., Chiryaeva O.G., Malysheva O.V., Dudkina V.S., Petrova L.I., Serebryakova E.A., Shabanova E.S. (2019). Reproductive History of a Woman With 8p and 18p Genetic Imbalance and Minor Phenotypic Abnormalities. Front. Genet..

[11] Li H., Handsaker B., Wysoker A., Fennell T., Ruan J., Homer N., Marth G., Abecasis G., Durbin R. (2009). 1000 Genome Project Data Processing Subgroup the Sequence Alignment/Map Format and SAMtools. Bioinforma.

[12] Kim S.K., Hannum G., Geis J., Tynan J., Hogg G., Zhao C., Jensen T.J., Mazloom A.R., Oeth P., Ehrich M. (2015). Determination of Fetal DNA Fraction from the Plasma of Pregnant Women Using Sequence Read Counts: Determination of Fetal DNA Fraction from the Plasma of Pregnant Women Using Sequence Read Counts. Prenat. Diagn..

[13] Johansson L.F., de Weerd H.A., de Boer E.N., van Dijk F., te Meerman G.J., Sijmons R.H., Sikkema-Raddatz B., Swertz M.A. (2018). NIPTeR: An R Package for Fast and Accurate Trisomy Prediction in Non-Invasive Prenatal Testing. BMC Bioinform..

[14] Efimova O.A., Pendina A.A., Krapivin M.I., Kopat V.V., Tikhonov A.V., Petrovskaia-Kaminskaia A.V., Navodnikova P.M., Talantova O.E., Glotov O.S., Baranov V.S. (2018). Inter-Cell and Inter-Chromosome Variability of 5-Hydroxymethylcytosine Patterns in Noncultured Human Embryonic and Extraembryonic Cells. Cytogenet. Genome Res..

[15] Pendina A.A., Efimova O.A., Tikhonov A.V., Chiryaeva O.G., Fedorova I.D., Koltsova A.S., Krapivin M.I., Parfenyev S.E., Kuznetzova T.V., Baranov V.S., Liehr T. (2017). Immunofluorescence Staining for Cytosine Modifications Like 5-Methylcytosine and Its Oxidative Derivatives and FISH. Fluorescence In Situ Hybridization (FISH).

[16] Koltsova A.S., Efimova O.A., Pendina A.A., Chiryaeva O.G., Osinovskaya N.S., Shved N.Y., Yarmolinskaya M.I., Polenov N.I., Kunitsa V.V., Sagurova Y.M. (2021). Uterine Leiomyomas with an Apparently Normal Karyotype Comprise Minor Heteroploid Subpopulations Differently Represented in Vivo and in Vitro. Cytogenet. Genome Res..

[17] Koltsova A.S., Efimova O.A., Malysheva O.V., Osinovskaya N.S., Liehr T., Al-Rikabi A., Shved N.Y., Sultanov I.Y., Chiryaeva O.G., Yarmolinskaya M.I. (2021). Cytogenomic Profile of Uterine Leiomyoma: In Vivo vs. In Vitro Comparison. Biomedicines.

[18] Wolstenholme J., White I., Sturgiss S., Carter J., Plant N., Goodship J.A. (2001). Maternal Uniparental Heterodisomy for Chromosome 2: Detection through “atypical” Maternal AFP/HCG Levels, with an Update on a Previous Case. Prenat. Diagn..

[19] Sifakis S., Staboulidou I., Maiz N., Velissariou V., Nicolaides K.H. (2010). Outcome of Pregnancies with Trisomy 2 Cells in Chorionic Villi. Prenat. Diagn..

[20] Chen C.-P., Su Y.-N., Chern S.-R., Chen Y.-T., Wu P.-S., Su J.-W., Pan C.-W., Wang W. (2012). Mosaic Trisomy 2 at Amniocentesis: Prenatal Diagnosis and Molecular Genetic Analysis. Taiwan. J. Obstet. Gynecol..

[21] Chen C.-P., Chen Y.-Y., Chern S.-R., Wu P.-S., Su J.-W., Chen Y.-T., Lee C.-C., Chen L.-F., Wang W. (2013). Prenatal Diagnosis of Mosaic Trisomy 2 Associated with Abnormal Maternal Serum Screening, Oligohydramnios, Intrauterine Growth Restriction, Ventricular Septal Defect, Preaxial Polydactyly, and Facial Dysmorphism. Taiwan. J. Obstet. Gynecol..

[22] Wang T., Lian J., Ren C., Huang H., Huang Y., Xu L., Zheng L., Cai C., Guo L. (2020). Prenatal Diagnosis of Mosaic Trisomy 2 and Literature Review. Mol. Cytogenet..

[23] Shaffer L.G., Langlois S., McCaskill C., Main D.M., Robinson W.P., Barrett I.J., Kalousek D.K. (1996). Analysis of Nine Pregnancies with Confined Placental Mosaicism for Trisomy 2. Prenat. Diagn..

[24] Chen C.-P., Ko T.-M., Chern S.-R., Wu P.-S., Chen Y.-N., Chen S.-W., Chen L.-F., Yang C.-W., Wang W. (2016). Prenatal Diagnosis of Low-Level Mosaicism for Trisomy 2 Associated with a Favorable Pregnancy Outcome. Taiwan. J. Obstet. Gynecol..

[25] Ariel I., Lerer I., Yagel S., Cohen R., Ben-Neriah Z., Abeliovich D. (1997). Trisomy 2: Confined Placental Mosaicism in a Fetus with Intrauterine Growth Retardation. Prenat. Diagn..

[26] Gibbons B., Cheng H.H., Yoong A.K., Brown S. (1997). Confined Placental Mosaicism for Trisomy 2 with Intrauterine Growth Retardation and Severe Oligohydramnios in the Absence of Uniparental Disomy in the Fetus. Prenat. Diagn..

[27] Roberts E., Dunlop J., Davis G.S., Churchill D., Davison E.V. (2003). A Further Case of Confined Placental Mosaicism for Trisomy 2 Associated with Adverse Pregnancy Outcome. Prenat. Diagn..

[28] Nagamatsu T., Kamei Y., Yamashita T., Fujii T., Kozuma S. (2014). Placental Abnormalities Detected by Ultrasonography in a Case of Confined Placental Mosaicism for Trisomy 2 with Severe Fetal Growth Restriction. J. Obstet. Gynaecol. Res..

[29] Robinson J., Stewart H., Moore L., Gaunt L. (1997). A Case of Mosaic Trisomy 2 Diagnosed at Amniocentesis in an Abnormal Fetus and Confirmed in Multiple Fetal Tissues. Clin. Genet..

[30] Seller M.J., Mazzaschi R., Ogilvie C.M., Mohammed S. (2004). A Trisomy 2 Fetus with Severe Neural Tube Defects and Other Abnormalities. Clin. Dysmorphol..

[31] Tuğ E., Karcaaltincaba D., Yirmibeş Karaoğuz M., Saat H., Özek A. (2017). Confirmation of the Prenatal Mosaic Trisomy 2 via Fetal USG and Cytogenetic Analyses. J. Matern.-Fetal Neonatal Med. Off. J. Eur. Assoc. Perinat. Med. Fed. Asia Ocean. Perinat. Soc. Int. Soc. Perinat. Obstet..

[32] Zhen L., Pan M., Li D.-Z. (2021). Pregnancies with Trisomy 2 Cells in Chorionic Villi: Ultrasound Determines the Outcome. Eur. J. Obstet. Gynecol. Reprod. Biol..

[33] Harrison K., Eisenger K., Anyane-Yeboa K., Brown S. (1995). Maternal Uniparental Disomy of Chromosome 2 in a Baby with Trisomy 2 Mosaicism in Amniotic Fluid Culture. Am. J. Med. Genet..

[34] Webb A.L., Sturgiss S., Warwicker P., Robson S.C., Goodship J.A., Wolstenholme J. (1996). Maternal Uniparental Disomy for Chromosome 2 in Association with Confined Placental Mosaicism for Trisomy 2 and Severe Intrauterine Growth Retardation. Prenat. Diagn..

[35] Hansen W.F., Bernard L.E., Langlois S., Rao K.W., Chescheir N.C., Aylsworth A.S., Smith D.I., Robinson W.P., Barrett I.J., Kalousek D.K. (1997). Maternal Uniparental Disomy of Chromosome 2 and Confined Placental Mosaicism for Trisomy 2 in a Fetus with Intrauterine Growth Restriction, Hypospadias, and Oligohydramnios. Prenat. Diagn..

[36] Liu S., Chang Q., Yang F., Xu Y., Jia B., Wu R., Li L., Yin A., Chen W., Huang F. (2023). Non-Invasive Prenatal Test Findings in 41,819 Pregnant Women: Results from a Clinical Laboratory in Southern China. Arch. Gynecol. Obstet..

[37] Ghiasi M., Armour C., Walker M., Shaver N., Bennett A., Little J. (2023). Issues Associated with Possible Implementation of Non-Invasive Prenatal Testing (NIPT) in First-tier Screening: A Rapid Scoping Review. Prenat. Diagn..

[38] Mortazavipour M.M., Mahdian R., Shahbazi S. (2022). The Current Applications of Cell-Free Fetal DNA in Prenatal Diagnosis of Single-Gene Diseases: A Review. Int. J. Reprod. Biomed. IJRM.

[39] Lannoo L., van Straaten K., Breckpot J., Brison N., De Catte L., Dimitriadou E., Legius E., Peeters H., Parijs I., Tsuiko O. (2022). Rare Autosomal Trisomies Detected by Non-Invasive Prenatal Testing: An Overview of Current Knowledge. Eur. J. Hum. Genet..

[40] Domaradzka J., Deperas M., Obersztyn E., Kucińska-Chahwan A., Brison N., Van Den Bogaert K., Roszkowski T., Kędzior M., Bartnik-Głaska M., Łuszczek A. (2021). A Placental Trisomy 2 Detected by NIPT Evolved in a Fetal Small Supernumerary Marker Chromosome (SSMC). Mol. Cytogenet..

